# Neuroprotective Effect of Maternal Resveratrol Supplementation in a Rat Model of Neonatal Hypoxia-Ischemia

**DOI:** 10.3389/fnins.2020.616824

**Published:** 2021-01-15

**Authors:** Ursule Dumont, Stéphane Sanchez, Cendrine Repond, Marie-Christine Beauvieux, Jean-François Chateil, Luc Pellerin, Anne-Karine Bouzier-Sore, Hélène Roumes

**Affiliations:** ^1^CRMSB, UMR 5536, CNRS/University of Bordeaux, Bordeaux, France; ^2^Département de Physiologie, University of Lausanne, Lausanne, Switzerland; ^3^CHU de Bordeaux, Place Amélie Raba Léon, Bordeaux, France; ^4^IRTOMIT, Inserm U1082, University of Poitiers, Poitiers, France

**Keywords:** polyphenol, resveratrol, neonatal hypoxia-ischemia, brain metabolism, MRI

## Abstract

Neonatal hypoxia-ischemia (nHI) is a major cause of death or subsequent disabilities in infants. Hypoxia-ischemia causes brain lesions, which are induced by a strong reduction in oxygen and nutrient supply. Hypothermia is the only validated beneficial intervention, but not all newborns respond to it and today no pharmacological treatment exists. Among possible therapeutic agents to test, *trans*-resveratrol is an interesting candidate as it has been reported to exhibit neuroprotective effects in some neurodegenerative diseases. This experimental study aimed to investigate a possible neuroprotection by resveratrol in rat nHI, when administered to the pregnant rat female, at a nutritional dose. Several groups of pregnant female rats were studied in which resveratrol was added to drinking water either during the last week of pregnancy, the first week of lactation, or both. Then, 7-day old pups underwent a hypoxic-ischemic event. Pups were followed longitudinally, using both MRI and behavioral testing. Finally, a last group was studied in which breastfeeding females were supplemented 1 week with resveratrol just after the hypoxic-ischemic event of the pups (to test the curative rather than the preventive effect). To decipher the molecular mechanisms of this neuroprotection, RT-qPCR and Western blots were also performed on pup brain samples. Data clearly indicated that when pregnant and/or breastfeeding females were supplemented with resveratrol, hypoxic-ischemic offspring brain lesions were significantly reduced. Moreover, maternal resveratrol supplementation allowed to reverse sensorimotor and cognitive deficits caused by the insult. The best recoveries were observed when resveratrol was administered during both gestation and lactation (2 weeks before the hypoxic-ischemic event in pups). Furthermore, neuroprotection was also observed in the curative group, but only at the latest stages examined. Our hypothesis is that resveratrol, in addition to the well-known neuroprotective benefits *via* the sirtuin’s pathway (antioxidant properties, inhibition of apoptosis), has an impact on brain metabolism, and more specifically on the astrocyte-neuron lactate shuttle (ANLS) as suggested by RT-qPCR and Western blot data, that contributes to the neuroprotective effects.

## Introduction

Neonatal hypoxia-ischemia (nHI) is the most common cause of perinatal brain injury, which may lead to neonatal death or irreversible damages like permanent physical disabilities, cerebral palsy and cognitive dysfunctions (Shankaran, 2012). It has a high global incidence of 1–8 per 1,000 live births, and remains a major cause of infantile mortality (Kurinczuk et al., 2010). It is caused by a massive reduction in cerebral blood flow and, therefore, oxygen and glucose supply to the brain. Today, the only applied treatment is moderate hypothermia (Gluckman et al., 2005), which cools down newborns to 33–34°C and is used in a time window of 72 h after the hypoxic-ischemic event (Bona et al., 1998). However, this treatment is only partly efficient and is unsuccessful for nearly half of the patients (Wu and Gonzalez, 2015). Therefore, there is an urgent need to find a complementary therapy or preventive treatment to further reduce the rate and severity of neurodevelopmental disabilities resulting from nHI.

According to the literature, evidence exists about the neuroprotective properties of *trans*-resveratrol (Bastianetto et al., 2015). Resveratrol (3,5,4′-trihydroxystilbene) is a natural polyphenol present at relatively high concentrations in peanuts, grape skin and seeds. This polyphenol possesses interesting biological properties such as anti-inflammatory (Das and Das, 2007), antioxidant (Gao et al., 2018) and anti-apoptotic activities (Bian et al., 2017). These activities may have promising potential therapeutic applications, as shown *in vivo* (Baur and Sinclair, 2006). Increasing evidence indicates that resveratrol may also play a role in the prevention of neurodegenerative diseases, such as Alzheimer’s or Parkinson’s disease (Sawda et al., 2017; Wicinski et al., 2020). Interestingly, blood-borne resveratrol can cross the blood–brain barrier (BBB) (Baur and Sinclair, 2006).

During the last decade, this polyphenol has shown a neuroprotective effect both as a *pre*- and *post*-treatment in the context of nHI (West et al., 2007; Karalis et al., 2011; Arteaga et al., 2015; Pan et al., 2016; Bian et al., 2017; Gao et al., 2018). Indeed, when administered just after the hypoxic-ischemic event [intraperitoneal injection (i.p.); 90 mg/kg], data indicated that resveratrol is neuroprotective as demonstrated by a strong reduction of the infarct volume and by the recovery of some behavioral functions (Karalis et al., 2011). This neuroprotection was also observed when resveratrol was administered before the hypoxic-ischemic event (Arteaga et al., 2015). When resveratrol was injected 10 min before the hypoxic-ischemic event (i.p.; 20 mg/kg; using the classical Rice-Vannucci postnatal day 7 (P7) pup model), a reduction in lesion volumes and an improvement of cognitive and motor functions (long-term assessment) were observed (Arteaga et al., 2015). The administration of resveratrol before the hypoxic-ischemic episode seems even more neuroprotective than when administered after.

Taking into account the fact that (i) maternal supplementation has an impact not only on pregnant women but also on the fetus (Nnam, 2015), (ii) resveratrol has neuroprotective properties and (iii) resveratrol and its metabolites can cross the placental barrier and the BBBs (Bourque et al., 2012; Shankaran, 2012), we aimed to evaluate the neuroprotective role of maternal resveratrol supplementation, at nutritional dose, in a context of nHI. The originality of our approach lies in the combination of (i) a transgenerational and nutritional approach, (ii) a longitudinal study of brain lesion evolution using non-invasive MRI associated to several behavioral tests, and (iii) a study of some gene and protein expressions in the brain of pups to try to decipher the molecular mechanisms of this neuroprotection. For this purpose, expression of a sirtuin (SIRT1), superoxide dismutase (SOD) and an antiapoptotic protein (Bcl2) was measured in brain samples, as well as of different key partners in brain metabolism, in particular, genes and proteins linked to the astrocyte-neuron lactate shuttle (ANLS) (Pellerin and Magistretti, 1994) such as the astrocytic glutamate transporters (GLT-1 and GLAST), the monocarboxylate transporters (MCT1 and MCT2), the lactate dehydrogenase isoforms (LDHa and LDHb) and the glial Na^+^/K^+^ATPase α_2_ isoform.

## Materials and Methods

### Drug Administration

All animal procedures were conducted in accordance with the Animal Experimentation Guidelines of the European Communities Council Directive of November 24, 1986 (86/609/EEC). Protocols met the ethical guidelines of the French Ministry of Agriculture and Forests and were approved by the local ethics committee (CEEA50). Resveratrol was purchased from Sigma (Sigma-Aldrich, France). Resveratrol stock solution was prepared by dissolving 100 mg of resveratrol in 2 ml of absolute ethanol (Sigma-Aldrich, France). A pregnant Wistar female rat drinks on average 50 ml water/day. For resveratrol supplementation, 4 μl of the resveratrol stock solution were added in 200 ml of drinking water, which results in a nutritional maternal consumption of 0.15 mg/kg/day. Resveratrol solutions in drinking bottles were prepared and changed every day. Because of resveratrol photosensitivity, drinking bottles were enwrapped in aluminum foil to avoid oxidation. 4 μl of absolute ethanol were added in drinking water (200 ml) for all sham groups during 2 weeks (last week of gestation + first week of breastfeeding).

### Experimental Groups

**FIGURE 1:**
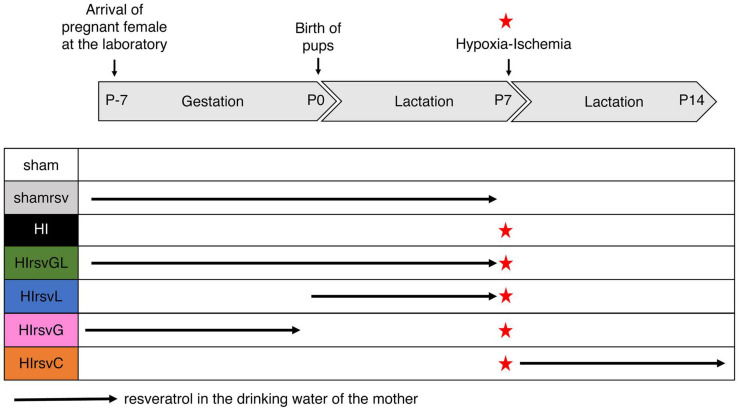
Experimental groups with different resveratrol regimens. Seven groups were studied: sham (water without any supplementation for the mother; no hypoxic-ischemic event for the pups), shamrsv (0.15 mg/kg/day of resveratrol in the drinking water of the mother during last week of gestation + first week of lactation; no hypoxic-ischemic event for pups), HI (water; insult at P7), HIrsvGL (0.15 mg/kg/day of resveratrol during last week of gestation + first week of lactation; insult at P7), HIrsvG (0.15 mg/kg/day of resveratrol during last week of gestation; insult at P7), HIrsvL (0.15 mg/kg/day of resveratrol during first week of lactation; insult at P7), HIrsvC (0.15 mg/kg/day of resveratrol during 1 week after the insult, performed at P7). Red star: hypoxic-ischemic event.

Pregnant Wistar female rats (Janvier Laboratories, France) were obtained at day 15 of gestation and kept on a 12 h/12 h light/dark cycle with A03 food (SAFE, Augy, France) and water *ad libitum*. Seven experimental groups were established according to the maternal supplementation plan (Figure 1).

Three control groups were first considered. The sham group for which there was no maternal resveratrol supplementation and in which pups did not undergo a hypoxic-ischemic episode but only carotid exposure (*n* = 18). The shamrsv group, for which resveratrol was added to the mother’s drinking water during the last week of gestation and the first week of lactation and in which pups did not undergo a hypoxic-ischemic episode (only carotid exposure). This control group was performed to ensure that resveratrol had no effect on healthy pups (see Supplementary Figure 1, *n* = 6). Finally, the HI group, without maternal resveratrol supplementation and in which pups underwent a hypoxic-ischemic event at P7 (*n* = 31). One preliminary study was performed on this HI group to ensure the reproducibility of the model (*n* = 11).

In a second step, four supplementation groups in which pups underwent a hypoxic-ischemic event at P7 were considered: pups from a pregnant female that received resveratrol supplementation in drinking water (i) both during the last week of gestation and the first week of lactation (gestation + lactation group; HIrsvGL group; *n* = 15), (ii) only the last week of gestation (gestation group; HIrsvG group; *n* = 15), (iii) only the first week of lactation (lactation group; HIrsvL; *n* = 15), or (iv) only the second week of lactation (just after the hypoxic-ischemic event and during 1 week; curative group; HIrsvC group; *n* = 15).

### Model of Neonatal Hypoxic-Ischemic Brain Injury

Cerebral hypoxia-ischemia was performed using the Rice-Vannucci model (Rice et al., 1981) as described previously (Dumont et al., 2019). Briefly, postnatal day 7 (P7) rat pups of both genders were used in the experiments (after checking the absence of statistical difference between males and females in our study, data from both genders were pooled). Pups weighing 15–19 g were anesthetized using isoflurane (4% for induction and 1.5% for maintenance). For ischemia, neck skin and muscles along an anterior midline were incised and the left common carotid artery was permanently ligated (surgery duration never exceeded 10 min per pup). After surgery, pups were placed in a heated atmosphere (33 ± 1°C) during 30 min in order to recover. Hypoxia was induced by placing animals during 2 h in a hypoxic chamber (Intensive Care Unit Warmer, Harvard Apparatus, Les Ulis, France, 8% O_2_, 92% N_2_). The hypoxic chamber temperature was maintained at 33 ± 1°C to ensure a pup rectal temperature of 35.5 ± 0.5°C (normothermia) and 80% humidity. For sham pups (sham and shamrsv groups), the left common carotid artery was just exposed (under the same anesthetic procedure as described above) and after 30 min of recovery, pups were kept separated from the mother in a heated atmosphere (33 ± 1°C, 2 h).

### Longitudinal Frame Acquisition of *in vivo* MRI

MRI acquisitions were performed 2 h 1/2 after the carotid artery ligation (P7), then 48 h after (P9) and 23 days later (P30), on a horizontal 4.7 T Biospec 47/50 system (Bruker, Ettlingen, Germany) equipped with a 6 cm BG6 gradient system (1000 mT/m, Δ = 20 ms, δ = 4 ms). Pups were anesthetized with isoflurane (4% for induction and 1.5% for maintenance). Breathing was monitored by a ventral pressure sensor and body temperature was maintained at 35.5 ± 0.5°C during acquisition with a water-heated MRI bed. Anatomical T2-weighted images of the brain were obtained using a Rapid Acquisition with Relaxation Enhancement (RARE) sequence with 20 axial slices (0.7 mm thick), echo time (TE) = 50 ms, repetition time (TR) = 3000 ms and a total duration of 4 min 48 s. Brain lesion volumes were assessed by Diffusion Weighted Imaging (DWI): 20 axial slices (0.7 mm thick), 30 directions, *b*-value 1000 s/mm^2^, TE = 24 ms, TR = 2 s, Δ = 8.11 ms, δ = 2.5 ms, total duration = 17 min 04 s. At P7, DWI was systematically performed 3 h after carotid ligation. MR-angiography was performed to image the cerebral arterial blood flow using a Time-of-Flight (TOF)-3D-Fast low angle shot (FLASH) sequence (axial slice, 20 mm thick, TE = 2.2 ms, TR = 13 ms, flip angle = 15°, total duration = 10 min 59 s) on pups presenting no lesion at P7 to ensure that the ligation was present (see Supplementary Figure 2). In two cases, pups presented no ligation and were excluded from the study.

### MRI Analysis

Measurements were performed with Paravision 6.0.1 software (Bruker BioSpin, Karlsruhe, Germany). Lesion volumes were measured at P7, P9 and P30 on the diffusion-weighted images. For each rat, for each slice and on the 20 adjacent slices per rat, two region-of-interests (ROI) were manually delineated to encompass the global brain area and injured area, respectively. Volume was then obtained by considering each slice thickness (0.7 mm). Lesion volumes were therefore expressed as a percentage of the total brain volume. In order to be free from any artifacts and to standardize the analyses, the threshold for detecting a lesion was set at 1% of the total volume of the brain. For this reason, only lesions larger than 1% of total brain volume were included in the study. The apparent diffusion coefficient (ADC) values (mm^2^/s) at P7 were obtained directly from the ADC maps produced by the software (Paravision 6.0.1) after DWI acquisition. ROIs were manually drawn on these maps such as delineating the brain lesion inside the cortex and hippocampus on the same slice, and the striatum on another appropriate slice as previously described (Dumont et al., 2019).

### Behavioral Tests

**FIGURE 2:**
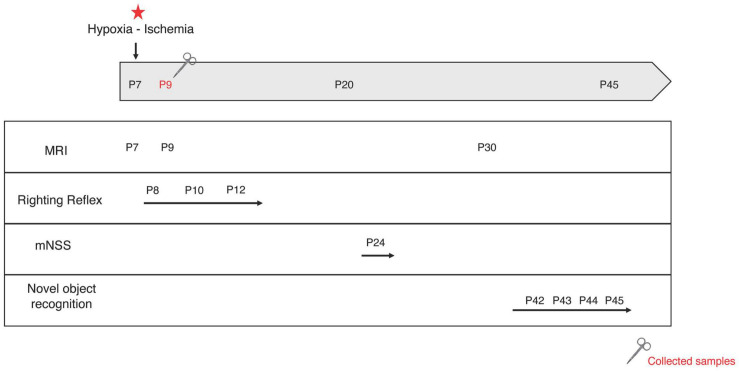
Experimental design. At postnatal day 7 (P7), the left common carotid artery was permanently ligated. Pups were exposed to a hypoxic environment (8% oxygen, 33°C, 2 h). MRI acquisitions were performed at P7, P9, and P30. Behavioral tests were performed at different time points: righting reflex at P8, P10, and P12; mNSS at P24 and novel object recognition test from P41 to P45. Some brains were collected for mRNA and protein analysis at P9. Red star: hypoxic-ischemic event.

The classical Rice-Vannucci model induces large ipsilateral cortical, hippocampal and striatal injuries (Ashwal et al., 2007), leading to sensorimotor (involving the cortex and striatum) and cognitive (involving the hippocampus) deficits (Lebedev et al., 2003). Cortical and striatal neuroprotection was assessed at short term with the righting reflex and later with the mNSS. Hippocampal neuroprotection was assessed using the novel object recognition test, allowing the evaluation of hippocampal-dependent long-term memory. Behavioral tests were performed on pups from P8 to P45 (Figure 2).

#### Righting Reflex (P8, P10, and P12)

Righting reflex was conducted on HI pups and on pups in groups that had completed their supplementation window (HIrsvGL, HIrsvG, and HIrsvL). Pups were turned on their back. The time required to turn over on all four paws was measured. Each pup was given three attempts and the meantime of the three trials was calculated.

#### Modified Neurological Severity Score (mNSS) (P24)

The mNSS test is a neurological scale widely used in the study of stroke in various models (Morris et al., 2010). The assessment of neurological disorders is based on a scale ranging from 0 to 18. This exam is composed of a series of motor (muscle condition, abnormal movement), sensory (visual, tactile or proprioceptive), reflex and balance tests (18 tests). One point is awarded if the animal fails to perform the task while no point is given if successful. Each rat was graded on the mNSS scale: the higher the score, the more impaired the sensorimotor functions (0–1: no impairment; 2–6: moderate impairments; 7–12: impairments; 13–18: severe impairments).

#### Novel Object Recognition Test (P42–P45)

The novel object recognition test is based on the natural tendency of rodents to explore novelty (Ennaceur and Delacour, 1988). Long-term memory was assessed in our task (Roumes et al., 2020). After a 10-min habituation period in the open field on day 1 (P42), pups were further allowed to freely explore two identical objects placed in opposite corners of the arena for 10 min on the next 2 days. On the last day (P45), one of the old objects was replaced by a new and different one, then pups were allowed to freely explore objects for 10 min. Durations of each object exploration were measured. The discrimination index (score between −1 and +1) was defined as the parameter for long-term memory evaluation:

$$I=\left(\frac{\text{time}\,\mathrm{spent}\,\mathrm{on}\,\mathrm{new}\,\mathrm{object}-\mathrm{time}\,\mathrm{spent}\,\mathrm{on}\,\mathrm{familiar}\,\mathrm{object}}{\text{time}\,\mathrm{spent}\,\mathrm{on}\,\mathrm{new}\,\mathrm{object}+\text{time}\,\mathrm{spent}\,\mathrm{on}\,\mathrm{familiar}\,\mathrm{object}}\right)$$

A positive score indicates more time spent with the new object. Closer to + 1 the score, lesser is memory alteration.

### Brain Sample Processing

Brains were collected at P9 from some pups in the supplementation group with the strongest neuroprotective effects (HIrsvGL group), as well as from some pups in the control and sham groups for further analysis by RT-qPCR, Western blots and histology. Cortical and striatal structures were collected from both hemispheres in all three groups. Hippocampi were collected from both hemispheres in sham and HIrsvGL groups, and only in the contralateral hemisphere in the HI group since this brain region was completely degenerated in the ipsilateral hemisphere.

#### RNA and Protein Extractions

P9 pups were lethally anesthetized with an intraperitoneal injection of Ketamine (100 mg/kg of Imalgene, Merial Boehring, Centravet Lapalisse) and Xylazine (20 mg/kg of Paxman, Virbac France, Centravet Lapalisse) then quickly decapitated. Brains were rapidly removed and placed on ice under RNAse free conditions. Each structure (cortex, hippocampus and striatum) of both ipsilateral (left) (when possible) and contralateral (right) hemispheres was quickly dissected. For RT-qPCR, each brain structure sample was stored in RNAlater and for Western blots it was stored in RIPA solution (Tampon Pierce^TM^ RIPA lysis buffer 1× + inhibitor proteases cocktail 10×, Thermo Fisher). Then, all samples were stored at −80°C. For the most injured brains (mainly in the HI group), the tissue loss caused by the infarct did not allow to collect all structures from the injured side especially the hippocampus in the HI group (data for hippocampus and striatum are shown in Supplementary Figures 3, 4).

#### Brain Removal for Nissl Staining

Postnatal day 9 pups (*n* = 3 per group) were deeply anesthetized with isoflurane. Intra-cardiac perfusion with PBS 1× was performed during 10 min (18 ml/min), followed by perfusion of a paraformaldehyde solution (PFA 4%, in PBS 1×) during 10 min (18 ml/min). Then, brains were quickly sampled and post-fixed in PFA 4% overnight (4°C), followed by cryoprotection in PBS 1×–Sucrose 20% (24 h, 4°C) and PBS 1×–Sucrose 30% (24 h, 4°C). Brains were frozen with nitrogen vapors and kept at −80°C until they were cut into sections (16 μm) with a cryostat.

### Real-Time Quantitative Polymerase Chain Reaction Analysis (RT-qPCR)

Total RNA was extracted and purified from each brain structure using RNeasy Protect Mini Kit (#74106, Qiagen, Hombrechtikon, Switzerland). For cDNA synthesis, 200 ng of total RNA were reverse transcribed using the Multiscribe Reverse protocol: 50 μM Random Hexamers and RT Buffer 10× were added to 200 ng of samples. Samples were incubated in a thermocycler (Biometra^®^, Thermocycler) 10 min at 65°C. Then, 10 mM of dNTP, MgCl_2_, RNase inhibitor and Multiscribe Reverse were added to the reaction mix and incubated in a thermocycler 10 min at 25°C, 45 min at 48°C, and 5 min at 95°C. Then, 1 μl of synthesized cDNA was subjected to RT-qPCR using the suitable primers (reverse and forward: 0.3 μM; Table 1) and SYBR Green PCR Master Mix (Applied Biosystems, Luzern, Switzerland) to perform the PCR reaction in a total reaction volume of 10 μl. Each sample was measured in triplicates. The RT-qPCR protocol was as follows: 2 ng of cDNA samples were incubated at 95°C for 3 min then 40 cycles of 3 s at 95°C and 20 s at 60°C. mRNA expression levels were determined with the StepOnePlus^TM^ Real-Time PCR System (Applied Biosystems, Luzern, Switzerland) with RPS29 (Ribosomal Protein S29, Microsynth, Balgach, Switzerland) mRNA used as endogenous control for normalization purposes (van de Looij et al., 2014). For data analysis, differences were calculated using the ΔΔCt method using the RPS29 gene as housekeeping gene.

**TABLE 1:** Forward and reverse oligonucleotide sequences of target gene primers for RPS 29, SIRT1, Bcl2, SOD2, MCT1, MCT2, GLT1, GLAST, Na^+^/K^+^ATPase, LDHa, and LDHb.

Genes	Forward 5′-3′	Reverse 5′-3′
RPS29	GGCTTTTAGGATGGAAGGGAC	TGAAGGTGACAGCAGTCGGTTG
SIRT1	CATACTCGCCACCTAACCTAT	AACCTCTGCCTCATCTACATTT
Bcl2	GAGTACCTGAACCGGCATCT	GAAATCAAACAGAGGTCGCA
SOD2	GAGCAAGGTCGCTTACAGA	CTCCCCAGTTGATTACATTC
MCT1	CTTGTGGCGTGATCCT	GTTTCGGATGTCTCGGG
MCT2	GCTCCGTATGCTAAGGAC	CGATAGTGACGAGCCC
GLT1	GAGCATTGGTGCAGCCAGTATT	GTTCTCATTCTATCCAGCAGCCAG
GLAST	CATCCAGGCCAACGAAACA	GAGTCTCCATGGCCTCTGACA
Na+/K+ ATPase	TGTGATTCTGGCTGAGAACG	CAAGTCAGCCCACTGCACTA
LDHa	TGGCCTCTCCGTGGCAGACT	CCCCCAGACCACCTCAACACAA
LDHb	AGACTGCCGTCCCGAACAA	ATCCACCAGGGCAAGCTCA

*The specificity of oligonucleotide primers was verified using the program BLAST (National Center for Biotechnology Information, Bethesda, MD, United States).*

### Western Blotting

Protein concentrations from cell lysates were determined with a Micro BCA Protein Assay Kit (Thermo Scientific Pierce BCA Protein Assay), according to the manufacturer’s recommendations. Protein concentration was adjusted in order to obtain the same concentration for each sample, using Laemmli Buffer 4×. Samples and molecular standards (PageRuler Prestained Protein Ladder, Thermo Fisher) were loaded on a SDS-PAGE 10% polyacrylamide gel electrophoresis (PAGE), and then proteins were transferred onto a nitrocellulose membrane. Transfer (gel to membrane) was performed by semi-dry transfer (Trans Blot Turbo transfer System Bio Rad). At the end of the transfer, membranes were blocked for 1 h, under agitation, at room temperature with TBS-T 1X (TBT-0,1% Triton x-100), 10% Milk and 1% BSA. Then, membranes were incubated for 12 h at 4°C under agitation with the appropriate primary antibody described in Table 2 (MCT1, MCT2, LDHa, and LDHb, 1:1000 and GLAST, 1:500). Memcode Reversible Protein Stain (Pierce) served as loading control. Blots were revealed by chemiluminescence (WesternBright ECL; Witec AG, Luzern, Switzerland) and imaged with a detection system (Chemidoc XRS+, Bio-Rad, Switzerland). Densitometric analysis of chemiluminescent signals was performed with the Image lab software using the total protein (Memcode) normalization procedure as recommended (Taylor et al., 2013).

**TABLE 2:** List of antibodies for Western blotting.

Anticorps	Sources	Identifier
Rabbit anti-MCTl	Homemade	Pierre et al., 2000
Rabbit anti-MCT2	Homemade	Pierre et al., 2000
Rabbit anti-LDHa	Cell signaling	2012S
Rabbit anti-LDHb	Sigma-Aldrich	AV48210
Rabbit anti-GLAST	Abcam	ab416

### Nissl Staining

Nissl staining was performed to estimate and compare cell death between the different conditions (Zhu et al., 2015), as previously described (Dumont et al., 2019). Briefly, 16 μm thick brain sections at P9 were stained with cresyl violet (0.5%, Sigma-Aldrich, France) for 10 min and washed in distilled water for 10 s. Sections were dehydrated in 95 and 100% ethanol (2 min each), defatted in xylene and mounted on coverslips in Depex mounting solution (Sigma Aldrich, Germany). Brain sections were observed using an optical microscope (Leica STP6000). Image J software (National institutes of Health, Bethesda, MD, United States)^1^ was used to count nuclei. In the software, the 8 bit image contrast was enhanced by 2%. The threshold was adjusted and the analyze particle tool was used to automatically count cell nuclei. For each condition, three different brains were analyzed. For each brain, two images were taken in the penumbra area, at the level of the cortex or the striatum.

### Statistical Analysis

MRI quantifications were blindly performed by two separate experimenters (intra-experimenter variability <1%; inter-experimenter variability <5%). Statistical analysis and graphs were performed using GraphPad Prism 7.00 software. All data were expressed as mean ± standard error of the mean (s.e.m). Number of pups is stated for each experiment within figure legends. Statistical significance of the differences between multiple groups was determined using One-Way ANOVA and Fischer’s LSD *post hoc* test (distribution of the data sets was tested using both Bartlett’s and Brown and Forsythe’s tests). Statistical significance was defined as *p* < 0.05 (^∗^*p* < 0.05, ^∗∗^*p* < 0.01, ^∗∗∗^*p* < 0.001, and ^****^p < 0.0001).

## Results

### Maternal Resveratrol Supplementation Reduces Hypoxia-Ischemia-Induced Brain Damages

**FIGURE 3:**
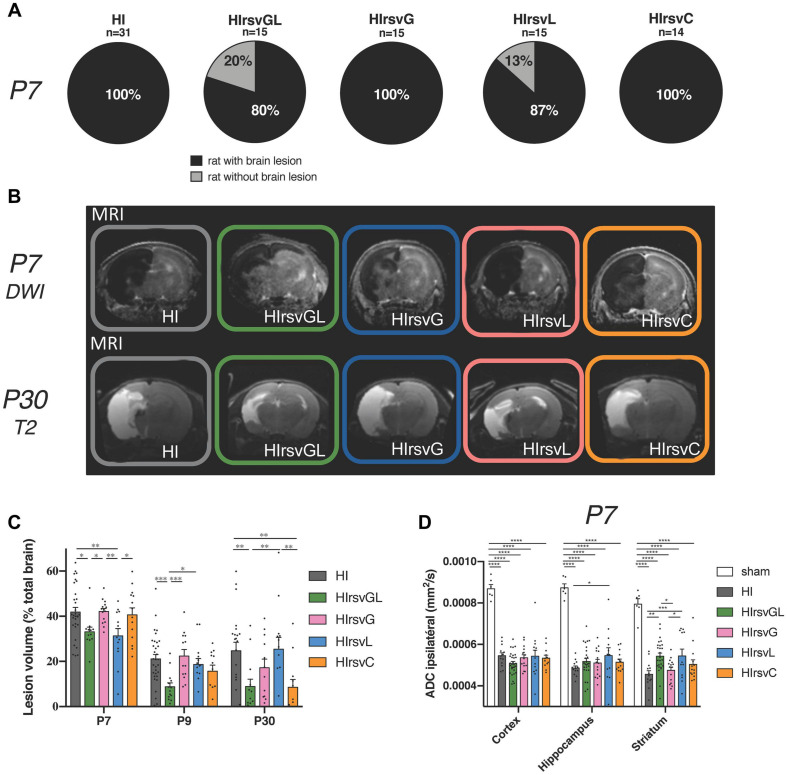
Visualization and quantification of brain lesion volume with or without resveratrol treatment after a hypoxic-ischemic event. **(A)** Percentage of P7 pups with brain lesion for HI, HIrsvGL, HIrsvG, HIrsvL, and HIrsvC groups, 3 h after injury. Pups with brain lesion volume <1% of the total brain volume were considered without brain lesion. **(B)** ADC maps obtained after DWI at P7 (top) and T2-weighted images at P30 (bottom) of pup brains for HI, HIrsvGL, HIrsvG, HIrsvL, and HIrsvC groups, acquired on a 4.7 T magnet. DWI was systematically performed 3 h after the common carotid artery ligation. Damage appeared as a hyposignal at P7 (decrease in ADC values due to the edema and cell swelling) and as a hypersignal at P30 (necrotic zone, water and cell death). **(C)** Lesion volumes (% total brain volume) at P7 (3 h after the common carotid artery ligation; *n* = 31, *n* = 12, *n* = 15, *n* = 13, and *n* = 15 for HI, HIrsvGL, HIrsvG, HIrsvL, and HIrsvC groups, respectively), at P9 (48 h after the hypoxic-ischemic event; *n* = 25, *n* = 14, *n* = 13, *n* = 14, and *n* = 12 for HI, HIrsvGL, HIrsvG, HIrsvL, and HIrsvC groups, respectively), and P30 (23 days after the hypoxic-ischemic event; *n* = 22, *n* = 13, *n* = 12, *n* = 13, and *n* = 12 for HI, HIrsvGL, HIrsvG, HIrsvL, and HIrsvC groups, respectively). **(D)** ADC values (mm^2^/s) in cortical, hippocampal and striatal lesions at P7. Results are mean values ± SEM. **(C,D)**: one-way analysis of variance (ANOVA) with Fisher’s LSD *post hoc* test. ^∗^: Significant difference between two groups (^∗^: *p* < 0.05, ^∗∗^: *p* < 0.01, ^∗∗∗^: *p* < 0.001, and ^****^: *p* < 0.0001).

At P7, all pups which underwent a hypoxic-ischemic event without treatment (Rice-Vannucci model; HI group) and all pups from the gestation (HIrsvG) and curative (HIrsvC) groups exhibited brain lesions (Figure 3A, first, third, and fifth pie charts). These lesions affected particularly the ipsilateral cortex, the hippocampus and the striatum. When resveratrol was administered to the mother either during the gestation (last week) + lactation period (first week) (HIrsvGL) or during the first week of lactation (HIrsvL), 20 and 13% of the pups had no MRI detectable brain lesion, respectively (Figure 3A; second and fourth pie charts). Only MRI data from P7 pups with MRI detectable brain damages were quantified in Figures 3C,D.

Diffusion- (P7) and T2- (P30) weighted images of pup brains from all groups are presented in Figure 3B. Brain lesions appear as a hyposignal at P7 (edema) whereas they appear as a hypersignal at P30 (necrotic zone). Brain lesion volumes (expressed as%, normalized to total brain volume) were measured for different time points and values are presented in Figure 3C. At P7, no significant difference was found between pups from the HI, HIrsvG, and HIrsvC groups (42 ± 2%, 42 ± 1%, and 41 ± 3%, respectively). Pups from the HIrsvGL and HIrsvL groups had significantly smaller lesions compared to those in the other hypoxic-ischemic groups (33 ± 2% and 31 ± 3%, respectively). At P9, pups from the HIrsvGL group showed the smallest brain lesion volumes compared to the other hypoxic-ischemic groups (9 ± 2% vs. 21 ± 2%, 22 ± 3%, 19 ± 3%, and 16 ± 3% for HI, HIrsvG, HIrsvL, and HIrsvC groups, respectively). At P30, no significant difference was detected in brain lesion volumes between the HI, HIrsvG, and HIrsvL groups (25 ± 4%, 17 ± 4%, and 25 ± 5%, respectively). The smallest brain lesion volumes were measured in the HIrsvGL and HIrsvC groups (9 ± 3% and 9 ± 3%, respectively). A decrease in brain lesion volume was detected in all groups within the first 48 h post-insult due to the spontaneous recovery of part of the damaged tissue (penumbra without cell death). The most extensive recovery was observed for the HIrsvGL group (mother with the longest resveratrol supplementation, before the hypoxic-ischemic event). In all groups, no statistical difference was found between P9 and P30.

Apparent diffusion coefficient values were measured in the cortex, hippocampus and striatum of all groups (Figure 3D). Independently of the considered structure, a significant drop in ADC values was detected in all hypoxic-ischemic groups compared to the sham group. Such a drop reflects the edema severity due to the water diffusion restriction induced by cell swelling. There was no significant difference between ADC values measured in hypoxic-ischemic groups in the cortex, while in the hippocampus, the HIrsvL group presented significantly higher ADC values compared to other hypoxic-ischemic groups, reflecting a less severe cytotoxic edema. The same increase in ADC values can be measured in the striatum of the HIrsvGL and HIrsvL groups compared to the HI and HIrsvG groups.

### Maternal Resveratrol Supplementation Preserves Cognitive and Sensorimotor Functions of Pups

**FIGURE 4:**
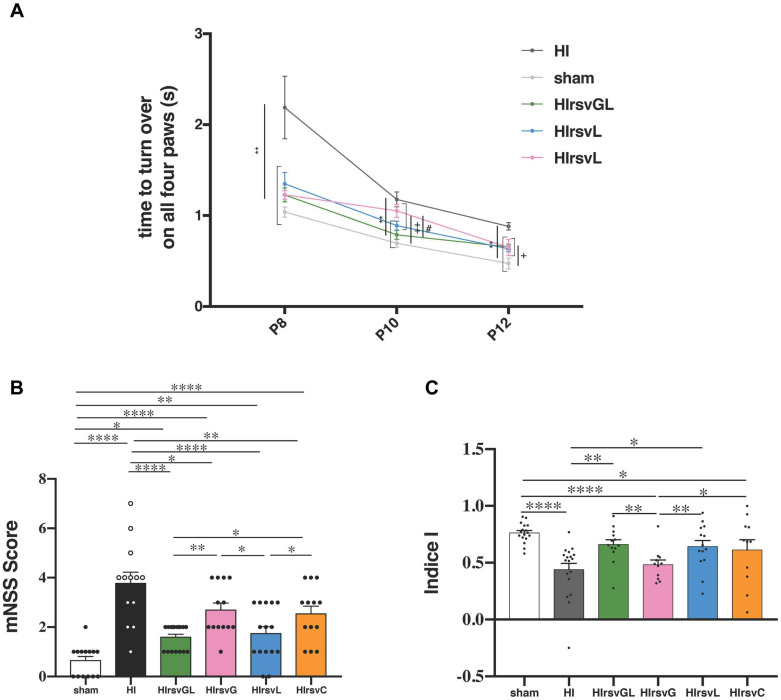
Behavioral investigations. **(A)** Righting reflex for sham, HI, HIrsvGL, HIrsvG, and HIrsvL groups (*n* = 23, *n* = 20, *n* = 17, *n* = 12, and *n* = 15, respectively) at P8, P10, and P12. **(B)** mNSS score at P24 for sham, HI, HIrsvGL, HIrsvG, HIrsvL, and HIrsvC groups (*n* = 14, *n* = 13, *n* = 17, *n* = 13, *n* = 15, and *n* = 13, respectively). Scoring between 0 and 18 points with: <1, no impairment; 1–6, moderate impairment; 7–12, impairment and 13–18, severe impairment. ^∗^: different from the HI group (^∗∗^: *p* < 0.01), +: different from the sham group (+: *p* < 0.05, ++: *p* < 0.01) and #: different from the HIrsvGL (*p* < 0.05). **(C)** Discrimination index of the novel object recognition test (performed at P45) for sham, HI, HIrsvGL, HIrsvG, HIrsvL, and HIrsvC groups (*n* = 17, *n* = 17, *n* = 13, *n* = 10, *n* = 14, and *n* = 11, respectively). Results are mean values ± SEM; one-way analysis of variance (ANOVA) with Fisher’s LSD *post hoc* test. ^∗^: Significant difference between two groups (^∗^: *p* < 0.05, ^∗∗^: *p* < 0.01, and ^****^: *p* < 0.0001).

Behavioral evaluations of pups in each group are shown in Figure 4. Righting reflex was performed from P8 to P12 (Figure 4A). At P8 and P12 the HI group presented a significantly longer time to turn back on their four paws compared to pups from the other groups. At P10 except from the HIrsvG group, all supplemented groups and the sham group performed significantly better than the HI group. Moreover, HIrsvG and HIrsvL groups presented a delay to reverse compared to the sham group. At P12, the sham group presented the best performances and the HI group, the worst, compared to the other hypoxic-ischemic groups.

Sensorimotor deficits were also assessed using the mNSS test (Figure 4B). The sham group showed no impairment (score <1; 0.6 ± 0.2) while the HI group had the highest neurological deficits (3.8 ± 0.4). Maternal resveratrol supplementation significantly decreased the neurological deficits, compared to the HI group (1.6 ± 0.1, 2.7 ± 0.3, 1.7 ± 0.3, and 2.5 ± 0.3 for the HIrsvGL, HIrsvG, HIrsvL, and HIrsvC groups, respectively). In the supplemented groups, pups from the HIrsvGL and HIrsvL groups presented the best scores.

Long-term memory was evaluated with the novel object recognition test at P45 (Figure 4C). The HI and HIrsvG groups showed a significantly lower discrimination index than sham pups (0.47 ± 0.06 and 0.48 ± 0.04, respectively vs. 0.76 ± 0.02 for sham group). No significant difference was detected between the sham, HIrsvGL, and HIrsvL groups. The HIrsvC group exhibited an intermediate discrimination index (0.61 ± 0.09) but with heterogeneous results.

### Maternal Resveratrol Supplementation Induces Changes in the Expression Pattern of Genes Involved in Sirtuin Signaling and Intercellular Metabolic Interactions

**FIGURE 5:**
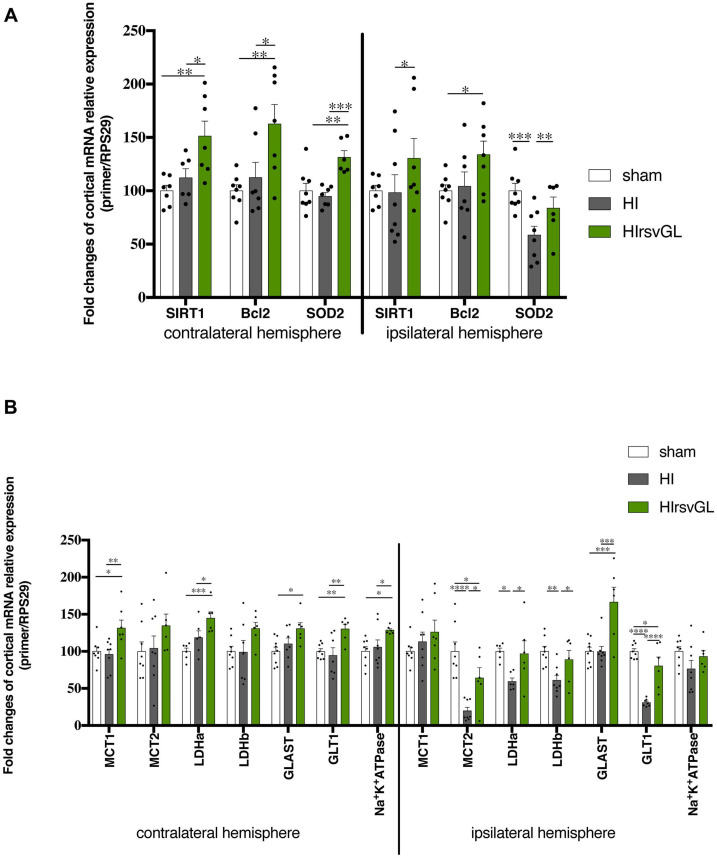
Impact of maternal resveratrol supplementation on cortical mRNA expression of some genes linked to signaling or metabolism. RT-qPCR performed at P9 on cortical samples from contralateral and ipsilateral hemispheres of sham, HIrsvGL, and HI groups (*n* = 6 for each group, hippocampus and striatum data, see Supplementary Figures 3, 4). **(A)** Signaling pathway: SIRT1, Bcl2, and SOD2 mRNA levels were quantified using appropriate primer sequences (see Table 1). **(B)** Metabolic pathway: MCT1, MCT2, LDHa, LDHb, GLAST, GLT1, and Na^+^/K^+^-ATPase α_2_ subunit mRNA levels were quantified using appropriate primer sequences (see Table 1). Results are mean values ± SEM and one-way analysis of variance (ANOVA) with Fisher’s LSD *post hoc* test. ^∗^: Significant difference between two groups (^∗^: *p* < 0.05, ^∗∗^: *p* < 0.01, ^∗∗∗^: *p* < 0.001, and ^****^: *p* < 0.0001).

Mechanisms involved in short-term neuroprotection by resveratrol were further evaluated using RT-qPCR on P9 cortical, hippocampal and striatal samples of pups from the HIrsvGL group (Figure 5 and Supplementary Figures 3, 4). For the HI group, no hippocampal tissue could be collected in the ipsilateral hemisphere (necrotic area), therefore no data was obtained for this structure on the injured side. Putative anti-apoptotic and antioxidant effects of resveratrol supplementation were assessed by quantification of SIRT1, Bcl2, and SOD2 mRNA expression in both contralateral (right) and ipsilateral (left) structures (cortex: Figure 5A, hippocampus and striatum: Supplementary Figures 3A, 4A, respectively). A possible impact of maternal supplementation on glutamate uptake and astrocyte-neuron lactate shuttling was explored by quantification of MCT1, MCT2, LDHa, LDHb, GLAST, GLT1, and Na^+^/K^+^ATPase α_2_ subunit mRNA expression in both undamaged and damaged structures (cortex: Figure 5B, hippocampus and striatum: Supplementary Figures 3B, 4B, respectively). Independently of the considered structure, maternal resveratrol supplementation significantly enhanced SIRT1 mRNA expression in all structures of the undamaged hemisphere compared to sham and HI groups (increases of 53 ± 13%, 45 ± 7%, and 36 ± 4%, compared to the sham group for cortical, hippocampal and striatal mRNA expressions, respectively) and only in the cortex of the ipsilateral hemisphere compared to the HI group. Whatever the considered structure, no significant difference in SIRT1 mRNA expression was measured between the sham and HI groups, neither in the ipsilateral nor in the contralateral hemisphere. Bcl-2 mRNA expression was increased by RSV maternal supplementation, in the cortex and the hippocampus of the contralateral hemisphere and in the ipsilateral cortex (relative Bcl-2 expression increase compared to the sham group: +67 ± 15%, +45 ± + 10%, and +28 ± 12% in the contralateral cortex and hippocampus and ipsilateral cortex, respectively). The hypoxic-ischemic event *per se* did not modulate Bcl2 mRNA expression. In addition, SOD2 mRNA content was higher in the undamaged cortex of the HIrsvGL group (relative SOD2 expression increase compared to the sham group: +31 ± 6%). While its expression was not altered in the uninjured hippocampus, it was reduced in the striatum on the uninjured side by maternal RSV supplementation compared to the other groups. In the damaged hemisphere, hypoxia-ischemia induced a decrease of SOD2 mRNA expression, which was reversed by maternal resveratrol supplementation, in the cortex as well as in the striatum.

Concerning genes linked to glutamate transport and lactate shuttling, the resveratrol supplementation enhanced the astrocytic transporter MCT1 mRNA expression in the undamaged cortex (relative MCT expression increase compared to the sham group: +31 ± 11%). In all hypoxic-ischemic groups, the expression of MCT2 mRNA was not modified in the uninjured structures. In the damaged cortex, the maternal supplementation with resveratrol partly counteracted the decrease in MCT2 mRNA expression induced by hypoxia-ischemia. The decrease in MCT2 expression was entirely counteracted in the striatum of the damaged hemisphere. The LDHa mRNA expression was enhanced by resveratrol supplementation in the cortex and hippocampus of the contralateral hemisphere, compared to the sham group (relative LDHa expression increase compared to the sham group: +45 and +53% in cortex and hippocampus, respectively). In the cortex of the damaged hemisphere, maternal supplementation counteracted the decrease in LDHa mRNA expression induced by hypoxia-ischemia. Concerning the LDHb gene, its mRNA expression in the contralateral hemisphere was increased in the cortex (relative LDHb expression increase compared to the sham and HI groups: +31%) and was restored in the striatum, while maternal resveratrol supplementation had no effect in the hippocampus. In the injured hemisphere, the decrease in LDHb mRNA induced by hypoxia-ischemia was counteracted only in the cortex. Concerning glutamate transporters, GLAST mRNA expression was only increased by maternal resveratrol supplementation in the cortex of both hemispheres. GLT1 mRNA expression was increased by maternal resveratrol supplementation in the cortex and the hippocampus of the contralateral hemisphere (relative GLT1 expression increase compared to the sham group: +30 and +32%, in the cortex and in the hippocampus, respectively). In the ipsilateral hemisphere, the decrease of its mRNA expression, induced by hypoxia-ischemia, was partially counteracted in the cortex by maternal resveratrol supplementation. Finally, concerning the expression of the Na^+^/K^+^ATPase α_2_ subunit mRNA, it was increased by maternal supplementation in the contralateral cortex and hippocampus (+29 and +31%, respectively, compared to the sham group). No statistical difference was detected in the ipsilateral hemisphere.

### Maternal Resveratrol Supplementation Increases Cortical Expression of Key Proteins Linked to the Astrocyte-Neuron Lactate Shuttle (ANLS)

**FIGURE 6:**
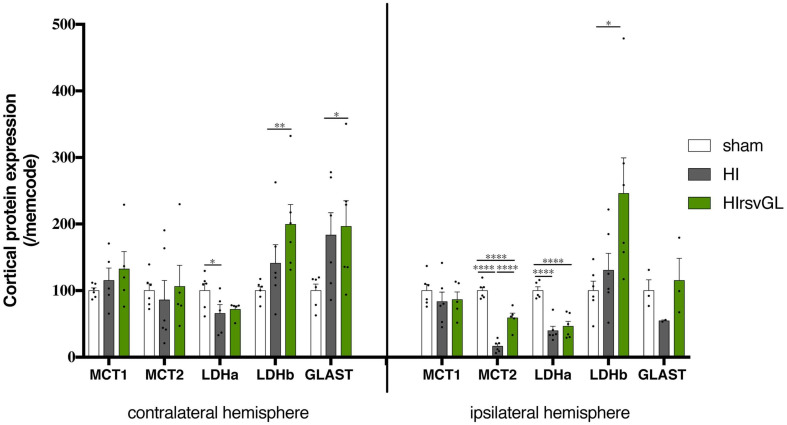
Impact of maternal resveratrol supplementation on expression of key ANLS proteins. Cortical expression levels of MCT1, MCT2, LDHa, LDHb, GLAST, GLT1, and the Na^+^/K^+^-ATPase α_2_ subunit proteins were quantified by Western blots (*n* = 6 for each group). Quantifications were normalized to Memcode staining. Results are mean values ± SEM and one-way analysis of variance (ANOVA) with Fisher’s LSD *post hoc* test. ^∗^: Significant difference between two groups (^∗^: *p* < 0.05, ^∗∗^: *p* < 0.01, and ^****^: *p* < 0.0001).

Results at the mRNA level prompted us to further investigate if protein expression of key elements of the ANLS was modified. MCT1 protein expression showed no statistically significant change in both cortices independently of the condition whereas maternal resveratrol supplementation partially restored MCT2 protein expression altered by the hypoxic-ischemic event only in the ipsilateral hemisphere (Figure 6). This was not the case for LDHa, which exhibited a drop in protein expression after hypoxia-ischemia for both damaged and undamaged cortices that was unaffected by the resveratrol treatment. However, maternal resveratrol supplementation increased LDHb protein levels in both cortices (+99 ± 30% and +146 ± 53% for contralateral and ipsilateral cortices compared to sham, respectively) although the hypoxic-ischemic event had no effect *per se*. Finally, for the astrocytic glutamate transporter GLAST, hypoxia-ischemia induced an up-regulation in the contralateral cortex (+96 ± 38%, compared to sham), which was unaffected by resveratrol supplementation. In the ipsilateral cortex, only a non-significant reduction in GLAST protein expression was observed after hypoxia-ischemia with a tendency to prevent this drop after the resveratrol treatment.

### Maternal Resveratrol Supplementation Reduces Cell Loss

**FIGURE 7:**
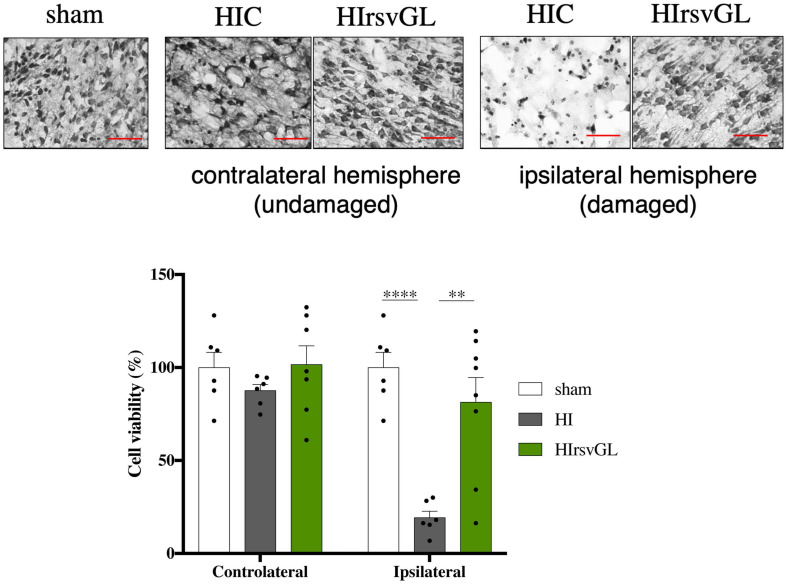
Evaluation of cell loss with or without resveratrol treatment after a hypoxic-ischemic event. Top panels: Nissl staining of 16 μm-thick cortical brain sections. Several neuro-morphological changes are observed in the pups from the HI group, with increased numbers of pyknotic cells and less Nissl stained nuclei. Maternal resveratrol supplementation preserved the number of Nissl stained nuclei (i.e., viable cells). Magnification ×20; scale bar: 100 μm. Bottom graph: Percentage of cell loss, based on the density of Nissl stained nuclei, for sham, HI, and HIrsvGL groups in either contralateral or ipsilateral cortex (*n* = 6). Results are mean values ± SEM, one-way analysis of variance (ANOVA) with Fisher’s LSD *post hoc* test. ^∗^: Significant difference between two groups (^∗∗^: *p* < 0.01 and ^****^: *p* < 0.0001).

Typical Nissl staining of cortical brain sections at P9 from the sham, HI and HIrsvGL groups are shown in Figure 7, top panels (striatum shown in Supplementary Figure 5, top panels). In the cortex and striatum, cell nuclei in the sham group displayed typical and homogenous morphologies. In the contralateral hemisphere, hypoxia-ischemia did not lead to significant cortical modification of Nissl staining but was responsible for a significant decrease in Nissl substance in the striatum (Figure 7 for cortex and Supplementary Figure 5 for striatum). In the ipsilateral hemisphere, several neuro-morphological changes were observed after the hypoxic-ischemic event in the HI group, with an increased in the number of pyknotic cells and a strong decrease in Nissl body content. Moreover, the number of Nissl stained nuclei was reduced in the HI group, in the injured cortex as well as in the injured striatum (−81 ± 4% and −42 ± 2%, in the damaged cortex and striatum, respectively, compared to sham group, Figure 7 bottom graph for cortex and Supplementary Figure 5 bottom graph for striatum). Such a decrease in Nissl stained nuclei was not observed in the uninjured and injured cortices of the HIrsvGL group as well as in the undamaged striatum of the HIrsvGL group. However, the number of Nissl stained nuclei could not be totally preserved in the striatum of HIrsvGL group (−31 ± 1%, compared to sham group).

## Discussion

Neonatal cerebral hypoxia-ischemia is an important cause of neonatal death and brain disorders with lasting deficiencies. Brain hypoxia-ischemia during the neonatal period leads to well-documented damages of the affected brain regions due to complex biochemical cascades (Kohlhauser et al., 1999). These activated processes lead to excitotoxicity, oxidative stress, deficiency in ATP-dependent pumps and cell death. In this study, we focused on maternal resveratrol supplementation, using a nutritional dose of resveratrol (0.15 mg/kg/d), which corresponds to the consumption of about 22 g of grapes (about thirty grape berries) per day for a pregnant woman (Dumont et al., 2020) and we followed brain lesions in P7-pups related to a hypoxic-ischemic event. In the HI group, the hypoxic-ischemic event induced severe lesions, since their volume represented around 40% of the total brain volume, as already characterized in this Rice-Vannucci model (Ashwal et al., 2007). Maternal resveratrol supplementation was tested during various time windows [the last week of gestation or/and the first week of lactation (preventive treatment) or the second week of lactation (curative treatment)]. Results indicated that the best neuroprotective effect was observed with the longest preventive supplementation period (HIrsvGL group), as already observed with piceatannol, an analog of resveratrol (Dumont et al., 2019). Indeed, MRI measurements at P7, 3 h post common carotid artery ligation, revealed a 21% decrease of brain lesion volume in the HIrsvGL group compared to the HI group. When resveratrol was administered during only 1 week (last week of gestation or first week of lactation), neuroprotection was not as strong as when it was administered during both weeks (HIrsvGL group). Concerning the incidence, 20% of the pups that underwent a hypoxic-ischemic insult in the HIrsvGL group had no brain lesion detected by DWI, whereas only 13% were without MRI detectable brain lesion in the HIrsvL group and 100% of the pups had brain lesions in the other groups. When looking at brain lesion volumes measured at short term, they were the smallest in the HIrsvGL group, and no statistical difference was found between the HI group (control) and the other groups treated during only 1 week. At long term, brain lesion volumes were the smallest in the HIrsvGL and HIrsvC groups, pointing out the long term effect of the curative treatment. Surprisingly, while a reduction in brain lesion volume was measured at short term between HIrsvL and HI groups, no difference was measured at long term. Finally, the mNSS test indicates that the smallest neurological deficits were observed in the HIrsvGL and HIrsvL groups while in the NOR test, pups in the HIrsvGL, HIrsvL, and HIrsvC groups showed the best memory performances and no difference was observed between pups in the HI and HIrsvG groups. When resveratrol supplementation lasted only 1 week before the hypoxic-ischemic event, the best results were obtained in the HIrsvL group compared to the HIrsvG group suggesting that the neuroprotection is more efficient when the breastfeeding female is under supplementation when the hypoxic-ischemic event occurs in the pups. Taken altogether, our data clearly indicate that the best neuroprotection was obtained in the HIrsvGL group, therefore when resveratrol was administered for a longer period prior to the hypoxic-ischemic insult. Therefore, to maximize the probability to observe significant modifications, mRNA and protein expression were analyzed only in this group.

### Nutritional Approach vs. Direct Treatment

Several publications have already reported that a direct pretreatment with resveratrol prevents neuronal injury in a similar context of nHI. In particular Arteaga et al. (2015) injected resveratrol (20 mg/kg; i.p.) in P7-pups just before hypoxia-ischemia (same Rice and Vannucci model) and they observed a 80% decrease in lesion volume 7 days after the insult (30 ± 2% for hypoxic-ischemic group compared to 5 ± 1% for resveratrol-treated group 7 days after the insult). Moreover, Gao et al. (2018) showed that a daily dose of resveratrol to pups (low dose 20 mg/kg or high dose 40 mg/kg; i.p.) for 1 week before hypoxia-ischemia could reduce the lesion size and cerebral edema in pups. They observed at P14, when resveratrol was administered 3 h post hypoxic-ischemic insult, a 40%-reduction of the lesion size for a “low” dose of resveratrol and a 50%-reduction for a high dose of resveratrol. Resveratrol had neuroprotective effects leading to functional benefits since, in addition to reduce the number of pups with lesion and decrease the infarct area, it improved behavioral deficits caused by hypoxia-ischemia. Indeed, our results showed a better motricity, as well as memory with resveratrol treatment, similarly to the results reported by Karalis et al. (2011) This study showed that direct administration of resveratrol to pups (90 mg/kg; i.p.) immediately after the hypoxic-ischemic insult (at P7) significantly reduced brain injuries as well as short- and long-term behavioral impairments. Strikingly, 24 h after the hypoxic-ischemic insult, resveratrol-treated pups exhibited better performance in righting reflex in this latter study, similarly to our observations.

Our study demonstrates a significant neuroprotection that is comparable to the one reported in the literature in a similar neonatal hypoxic-ischemic model but after a direct treatment of pups. However, our approach is certainly more likely to be acceptable for a transfer to the clinic since the mode of administration of the treatment (in pregnant and/or breastfeeding female’s drinking water compared to i.p. injection) is less invasive and the dose (our daily dose of 0.15 mg/kg compared to 20–100 mg/kg) is within a reasonable nutritional range. An important point raised by the comparison between the literature and our results, is that the best results are observed when resveratrol is present at the time of the hypoxic-ischemic event. Indeed, in the HIrsvG group (resveratrol treatment was stopped 7 days before the hypoxic-ischemic insult), the neuroprotection was not as efficient as the one observed for HIrsvGL or HIrsvL groups, suggesting that the closest to the hypoxic-ischemic event the supplementation, the more neuroprotective.

### Correlation Between Lesion Size and Behavioral Performance

MRI is a powerful technique that allows a longitudinal follow-up of animals. Such a non-invasive technique could thus be coupled with behavioral tests at different ages to correlate with cognitive performance. Indeed, images alone are not necessarily predictive of brain functions, since rat pups have a high neuronal plasticity, which allows them to spontaneously recover some brain capacities (Wittmann et al., 2004). Moreover, MRI does not allow to always detect brain lesions, especially when they are too small or diffused. Therefore, performing behavioral tests become mandatory. Pups from the HIrsvGL group exhibited the smallest lesion volumes and the best performances in all tests compared to the other groups. These results are in accordance with a previous study showing a greater reduction in brain damages and a better preservation of behavioral abilities after maternal piceatannol supplementation during gestation and breastfeeding than during breastfeeding alone (Dumont et al., 2019). Interestingly, pups from the HIrsvL group showed the same performance level than sham pups and pups from the HIrsvGL pups in behavioral tests, while their brain lesion volumes were bigger than the one measured in pups from the HIrsvGL group (at any time point), and not statistically different from the brain lesion volumes of pups from the HI group. These results clearly indicate that (i) MRI alone just after the insult may not be accurately predictive of eventual brain function impairments and (ii) behavioral tests are necessary to evaluate the potential neuroprotection of a tested treatment. Concerning the time window for maternal resveratrol supplementation, data (MRI and behavior) indicated that when the resveratrol supplementation was stopped 1 week before the neonatal hypoxic-ischemic event, the neuroprotective effect was the lowest. When resveratrol was present only during the last week of gestation (HIrsvG), pups displayed a significant decrease in lesion size only at P30 and minimal recovery of short-term behavioral performances (righting reflex and mNSS scores). However, this neuroprotection was not sufficient to protect the hippocampus since long-term memory was not preserved. These data could be explained by the fact that the CA1 area of the hippocampus is usually the most vulnerable structure, followed by the neocortex and then by the striatum (Smith et al., 1984).

### Striatum-Specific Neuroprotective Effect

A hypoxic-ischemic insult leads to a deficiency in oxygen and glucose, inducing a decrease in ATP production and an alteration of all energetically dependent mechanisms. ATP-dependent pumps are no longer efficient, which leads to an ionic imbalance and a cellular water influx responsible for cell swelling and, at a larger scale, to cytotoxic edema (Badaut et al., 2011). ADC values, which decrease during the first hours after the insult due to water movement restriction linked to the edema, were measured by DWI, in the three most affected structures (cortex, hippocampus and striatum). A decrease in ADC values was measured, as observed in previous studies (Miyasaka et al., 2000; McKinstry et al., 2002). However, this decrease was slightly lower in the striatum of pups from the HIrsvGL and HIrsvL groups, indicating that cytotoxic edema was less severe. This slight neuroprotective effect in pups has already been shown in another study, using the same model of nHI, with lactate as a neuroprotective agent (Roumes et al., 2020). This striatal-specific neuroprotective effect was also observed after a middle cerebral artery occlusion in the mouse, in which lactate injection led to a significant reduction of the lesion size, which was more pronounced in the striatum (Berthet et al., 2009). Indeed, Berthet et al. (2009) in order to understand why lactate protected mainly the striatum, measured lactate concentrations in each structure after lactate injection in healthy mice by MR spectroscopy; they showed that the lactate concentration was higher in the striatum. In addition, another study indicated that resveratrol decreased anoxia-induced dopamine release and appeared as a promising agent to improve the alterations occurring under anoxic-ischemic conditions in striatal sections by protecting dopaminergic neurons (Gursoy and Buyukuysal, 2008). Since dopamine is implicated in the development of an ischemic lesion in the striatum, a reduction of dopamine release may protect this structure against ischemic lesions (Globus et al., 1987).

### Curative Treatment

Although maternal resveratrol supplementation administered before the hypoxic-ischemic event in pups was neuroprotective, we also wanted to test its potential curative effect. Therefore, a HIrsvC group was created, in which resveratrol was present in the drinking water of the breastfeeding female just after the neonatal hypoxic-ischemic event. Interestingly, even if administered after the insult, pups from the HIrsvC group had better mNSS scores and performed better in the novel object recognition test than pups from the HI group. Such a curative effect of resveratrol was also observed in the same context (and same model) but with a direct treatment (Pan et al., 2016). Indeed, 3 doses of resveratrol (100 mk/kg; i.p.) injected at 0, 8, and 18 h reduced the lesion sizes as well as the expression levels of key inflammatory factors and pro-apoptotic molecules. These data confirm that (i) MRI alone just after the insult is not a predictive technique of the sensory-cognitive-motor functions and (ii) it is necessary to evaluate the effectiveness of a therapeutic approach using a long-term evaluation.

### Involvement of the “Classical” Sirtuin Pathway

To further understand the basis of these effects, we aimed at deciphering the molecular mechanisms involved in resveratrol neuroprotection. It was shown that resveratrol could enter the cell by endocytosis *via* lipid rafts (Delmas et al., 2013; Neves et al., 2016), which are present on the plasma membranes of both neurons (Tsui-Pierchala et al., 2002) and astrocytes (Miller et al., 2020). However, once inside the cell, its mode of action is far from being fully understood. For this purpose, only pup’s brains from the most effective supplementation regimen (HIrsvGL group) were analyzed in parallel with brains from sham and HI groups. As a first step, we focused on some targets from a signaling pathway, which is known to be regulated by resveratrol [SIRT1 (Lagouge et al., 2006), SOD (Toader et al., 2013), Bcl2 (Pan et al., 2016)]. Hypoxia-ischemia is characterized by two consecutive traumatic events: hypoxia-ischemia itself and the reperfusion period, which induces oxidative stress and ROS production (Ten and Starkov, 2012). This increase in oxidative stress leads to a dysfunction of the Na^+^/K^+^-ATPase, brain edema and BBB disruption (Himadri et al., 2010). The Na^+^/K^+^-ATPase plays a major role in the maintenance of intracellular electrolyte homeostasis and has been shown to be particularly sensitive to ROS-induced damages (Lima et al., 2009). In response to this insult, SOD, by stimulating the conversion of superoxide to hydrogen peroxide, is one of the main antioxidant defense systems to scavenge ROS (Wang et al., 2018). Studies have described that the expression of SOD is regulated by SIRT1 through deacetylation of the transcription factor FoxO3a and the transcriptional coactivator peroxisome proliferator-activated receptor γ-coactivator 1α (PGC-1α) (Olmos et al., 2013). In our model, the neonatal insult induced a decrease in SOD expression, which was partially counteracted by maternal resveratrol supplementation, which also enhanced SIRT1 levels. Our results agree with previous data reported by Shin et al. who showed that resveratrol protected from ischemic stroke by upregulating the SIRT1-PGC-1α signaling pathway leading to an antioxidant effect under ischemic stress (Shin et al., 2012). In another study, using a P14-neonatal rat hypoxic-ischemic model, direct pre-treatment with resveratrol (20 or 40 mg/kg, for 7 consecutive days before hypoxic-ischemic induction) was shown to alleviate oxidative stress, in part by increasing SOD expression just after the hypoxic-ischemic event (Gao et al., 2018). Likewise, in an adult rat model of transient global cerebral ischemia, it has been reported that resveratrol (30 mg/kg; i.p. injections 7 days prior to ischemia) is neuroprotective and restores Na^+^/K^+^-ATPase activity in the cortex and hippocampus back to normal levels (Simao et al., 2011). Activation of SIRT1 signaling during nHI by resveratrol was paralleled by an increased expression of Bcl2. A SIRT1-dependent increase in Bcl2 has also been reported in a stroke context after curcumin pretreatment and has been linked to anti-inflammatory properties (Miao et al., 2016). Similarly, in oxygen–glucose deprivation (OGD) injury models (primary cortical neurons), an upregulation of SIRT1 caused an increase in the anti-apoptotic Bcl2 expression and a decrease in the pro-apoptotic cleaved caspase-3 expression (Yan et al., 2013). In our study, the increase of SIRT1, Bcl2, and SOD2 expression was confirmed, as shown in previous studies, which explored the direct impact of resveratrol. These data confirm that our transgenerational and nutritional approach is as efficient as a direct treatment and that similar upregulations of SIRT1, Bcl2, and SOD2 are observed in pup’s brains when low doses of resveratrol were administered to the mother compared to a direct administration of much higher doses.

### Impact of Maternal Resveratrol Supplementation on Neonatal Brain Metabolism

In a second step, we looked at the potential impact of maternal resveratrol supplementation on neonatal brain metabolism. Indeed, only a few studies have investigated the effect of resveratrol on cerebral metabolism under physiological or pathological conditions, and none looked at it in a transgenerational context. In a previous study, it was observed that transient forebrain ischemia in adult rat led to a decrease in GLT1 expression (Girbovan and Plamondon, 2015), the major astrocytic glutamate transporter. The decrease in astrocytic glutamate uptake induced by hypoxia-ischemia is responsible for glutamatergic excitotoxicity, which is a major cause of neuronal death. This decrease was reversed by resveratrol pretreatment (1 or 10 mg/kg dose; i.p.) (Girbovan and Plamondon, 2015). In our model, hypoxia-ischemia also induced a decrease in astrocytic GLT1 mRNA content in the damaged hemisphere, which was counteracted by maternal resveratrol supplementation. GLAST mRNA content was not downregulated by the neonatal hypoxic-ischemic event, but was upregulated by maternal resveratrol treatment (in both hemispheres). This upregulation of glutamate transporter mRNA expression by resveratrol may lead to a decrease in glutamatergic excitotoxicity promoting neuronal survival, as supported by the Nissl staining. Astrocytic glutamate uptake is also implicated in astrocyte-neuron metabolic interactions. Indeed, astrocytic glutamate reuptake (3Na^+^/glutamate co-transport) after neuronal activity has been shown to activate astrocytic glycolysis (through the Na^+^/K^+^-ATPase activation) and lactate production, which is transferred to neurons and used as a neuronal oxidative fuel (Pellerin and Magistretti, 1994). This lactate shuttling process is named ANLS for astrocyte-neuron lactate shuttle. Lactate is shuttled from astrocytes to neurons *via* monocarboxylate transporters (MCTs), with different isoforms exhibiting a preferential cellular localization (MCT2 on neurons, MCT1 on astrocytes and endothelial cells). In previous studies, we have shown that (i) resveratrol can activate glycolysis (*ex vivo* NMR spectroscopy study on perfused liver) (Beauvieux et al., 2013) and, more recently, (ii) that lactate is neuroprotective after a neonatal hypoxic-ischemic event (Roumes et al., 2020). Taken altogether, our results lead us to hypothesize that resveratrol supplementation, in addition to act through classical signaling pathways, may impact other metabolic pathways, and more particularly can modulate the expression of key partners involved in ANLS. Yet, our hypothesis is supported by the increase in mRNA levels of several key elements involved in the ANLS: the MCTs, the lactate dehydrogenase (LDH) isoforms (that converts pyruvate into lactate and *vice versa*), the astrocytic glutamate transporters (GLAST and GLT1) and the α_2_ subunit of the Na^+^/K^+^-ATPase. In the context of hypoxia-ischemia, during the latent phase, lactate may act as an alternative fuel to counteract the energy deficit that occurred during the hypoxic-ischemic event. This is consistent with the increased expression of the LDHb isoform (which favors the conversion of lactate into pyruvate and therefore favors lactate consumption) at the protein level, and not the LDHa isoform (which favors pyruvate into lactate conversion, and therefore favors lactate production). There is a selective distribution of these two subunits of LDH, the isoenzyme LDH-5 (4 LDHa subunits) is more present in glycolytic tissues while the isoenzyme LDH-1 (4 LDHb subunits) is more predominant in oxidative tissues (Markert et al., 1975). Another study has shown that neurons have a strong content in LDHb-enriched isoenzymes while astrocytes have more LDHa-enriched isoenzymes (Bittar et al., 1996). The metabolic use of lactate as an energetic substrate for neurons would then allow to spare glucose for other important cellular repair mechanisms, such as the pentose phosphate pathway (PPP), which activity is crucial for the production of reduced glutathione, a ROS scavenger (Morken et al., 2014). PPP has a critical role in neuronal survival during cerebral ischemia/reperfusion as it produces crucial antioxidant molecules in order to reduce the oxidative stress and inflammation (Cao et al., 2017). Finally, lactate may have a neuroprotective effect through reducing excitotoxicity. Indeed, it was recently shown on hippocampal slices that lactate in the medium can reduce neurotransmitter content at both excitatory and inhibitory synapses, thus limiting excitotoxicity, which is present during hypoxia-ischemia (Hollnagel et al., 2020). To conclude, we can hypothesize that resveratrol, in addition to its antioxidant, anti-apoptotic and anti-inflammatory properties, may also be neuroprotective by targeting key partners of the ANLS. However, a direct or indirect effect of resveratrol on brain metabolism and excitotoxicity still need to be further explored. In addition, we can not exclude a direct effect of resveratrol on the activities of some of these proteins [such as GLAST and GLT1, which are redox sensitive (Volterra et al., 1994; Hansel et al., 2014)].

### Limitations of the Study

Although this study is the first one that shows a regulation by resveratrol of genes linked to brain metabolism in pups after maternal supplementation, it presents limitations since our interpretation was based mainly on cortical mRNA levels and ANLS-linked protein expression levels (but not activities) at only one time point. For some proteins, the expression levels did not vary completely in accordance with their mRNA levels. However, since the impact of a hypoxia-ischemia episode and the effect of resveratrol on mRNA and protein levels might not follow the same time course, a more detailed study involving several time points (and in the different brain areas) would be necessary to clarify these issues.

## Conclusion

**FIGURE 8:**
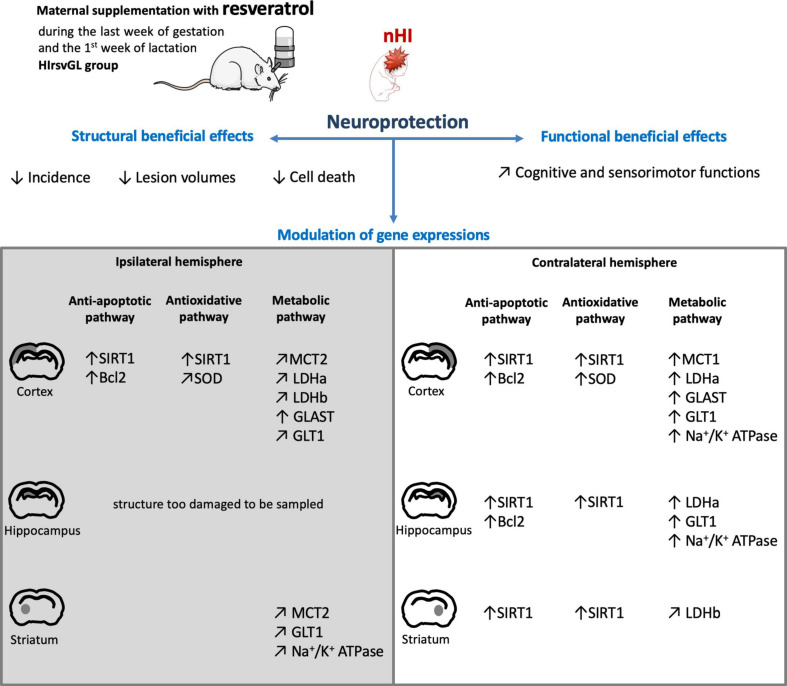
Scheme summarizing the neuroprotection on hypoxic-ischemic pups after 2 weeks of maternal resveratrol supplementation. Scheme illustrating the neuroprotective effects of maternal resveratrol supplementation (during the last week of gestation and the first week of lactation) on pup’s (i) brain structures, (ii) cognitive and sensorimotor functions and (iii) gene expression, linked to the anti-apoptotic, antioxidant and metabolic pathways. ↑: increased compared to the sham group; ↗: restored to the expression level of the sham group.

The originality of our study was to combine different techniques (MRI, behavioral investigations, histology, gene and protein expression analyses) to characterize brain lesions and their functional consequences, and to decipher the molecular mechanisms behind this neuroprotection. Based on behavioral data, we can rank the different supplementation time-windows as followed: gestation + lactation > lactation ≅ curative > gestation. A scheme that summarizes the impact of the most effective supplementation on hypoxic-ischemic pups is presented in Figure 8. This 2 weeks-maternal supplementation allows to decrease the incidence and the volumes of brain lesions detected by MRI, the extent of cell loss and, more importantly, to preserve motor and cognitive abilities. Furthermore, in addition to the classical sirtuin pathway, this study shows for the first time, that resveratrol may modulate brain metabolism by upregulating some key components of the ANLS. Such a modulation could participate to the beneficial effect of resveratrol. Of note, our proposed nutritional and transgenerational approach to administer resveratrol could be of great interest in the search for a realistic and easily implementable neuroprotective strategy against the deleterious effects induced by nHI.

## Data Availability Statement

The raw data supporting the conclusions of this article will be made available by the authors, without undue reservation.

## Ethics Statement

The animal study was reviewed and approved by Bordeaux Ethic Committee.

## Author Contributions

UD acquired and analyzed the data and contributed to the final drafting of the manuscript. SS produced the hypoxic-ischemic neonate model. CR participated to Western blot and RTqPCR experiments. M-CB and J-FC contributed to the final drafting of the manuscript. LP contributed to the design of the experiments and wrote the manuscript. A-KB-S and HR conceived, designed, acquired, and analyzed the data and wrote the manuscript. All authors contributed to the article and approved the submitted version.

## Conflict of Interest

The authors declare that the research was conducted in the absence of any commercial or financial relationships that could be construed as a potential conflict of interest.

## Footnotes

1 https://imagej.nih.gov/ij/download.html
