# Supplementary material for: Neuroprotective Effect of Maternal Resveratrol Supplementation in a Rat Model of Neonatal Hypoxia-Ischemia
Source: Front Neurosci. 2021 Jan 15;14:616824. doi: 10.3389/fnins.2020.616824 (PMC7844160; doi:10.3389/fnins.2020.616824)
Supplement: Supplementary file 1 [file Table_1.docx]

Supplementary Material

# Supplementary Figures

**Supplementary Figure 1. Maternal resveratrol supplementation had no deleterious effect on healthy pups.**

(A) ADC values (mm^2^/s) in cortical, hippocampal and striatal structures or lesions at P7 for sham, shamrsv and HI. (B) Righting reflex for sham, shamrsv and HI pups at P8, P10 and P12. (C) mNSS score at P24 for sham, shamrsv and HI pups; scoring between 0 and 18 points corresponds to: <1, no impairment; 1-6, moderate impairment; 7-12, impairment and 13-18, severe impairment (n= 14, n= 8 and n=13, respectively). (D) Discrimination index of the novel object recognition test (performed at P45) for sham, shamrsv and HI pups (n=17, n=6 and n=18, respectively). Results are mean values ± SEM and one-way analysis of variance (ANOVA) with Fisher’s LSD post-hoc test. *: Significant difference between two groups (*: p<0.05 and ****: p<0.0001).

**Supplementary Figure 2. Typical cerebral angiographic images of P7 pups that underwent or not left artery carotid ligation.**

A: typical angiography in sham pups; red arrow: location of the left common carotid artery. All HI-pups considered in this study had typical angiography shown in B.

**Supplementary Figure 3. Impact of maternal RSV supplementation on hippocampal mRNA expression of some genes linked to signaling or metabolism**

RT-qPCR was performed on hippocampal samples at P9 from contralateral and ipsilateral hemispheres of pups from sham, HIrsvGL and HI groups (n=6 for each group). (A) Signaling pathway: SIRT1, Bcl2 and SOD2 mRNA levels were quantified using appropriate primer sequences (see Table 1). (B) Metabolic pathway: MCT1, MCT2, LDHa, LDHb, GLAST, GLT1 and Na^+^/K^+^-ATPase α_2_ subunit mRNA levels were quantified using appropriate primer sequences (see Table 1). Results are mean values ± SEM and one-way analysis of variance (ANOVA) with Fisher’s LSD post-hoc test. *: Significant difference between two groups (*: p < 0.05, **: p < 0.01, ***: p < 0.001 and ****: p < 0.0001).

**Supplementary Figure 4. Impact of maternal RSV supplementation on striatal mRNA expression of some genes linked to signaling or metabolism**

RT-qPCR was performed on striatal samples at P9 from contralateral and ipsilateral hemispheres of pups from sham, HIrsvGL and HI groups (n=6 for each group). (A) Signaling pathway: SIRT1, Bcl2 and SOD2 mRNA levels were quantified using appropriate primer sequences (see Table 1). (B) Metabolic pathway: MCT1, MCT2, LDHa, LDHb, GLAST, GLT1 and Na^+^/K^+^-ATPase α_2_ subunit mRNA levels were quantified using appropriate primer sequences (see Table 1). Results are mean values ± SEM and one-way analysis of variance (ANOVA) with Fisher’s LSD post-hoc test. *: Significant difference between two groups (*: p < 0.05, **: p < 0.01, ***: p < 0.001 and ****: p < 0.0001).

**Supplementary Figure 5. Evaluation of striatal cell death.**

Top panels: Nissl staining of 16µm-thick striatal brain sections at P9. Magnification ×20; scale bar: 100 µm. Bottom graph: Percentage of cell death in striatum of pups from sham, HI and HIrsvGL groups in both contralateral and ipsilateral hemispheres (n=6). Results are mean values ± SEM, one-way analysis of variance (ANOVA) with Fisher’s LSD post-hoc test. *: Significant difference between two groups (*: p < 0.05 and ****: p < 0.0001).

**
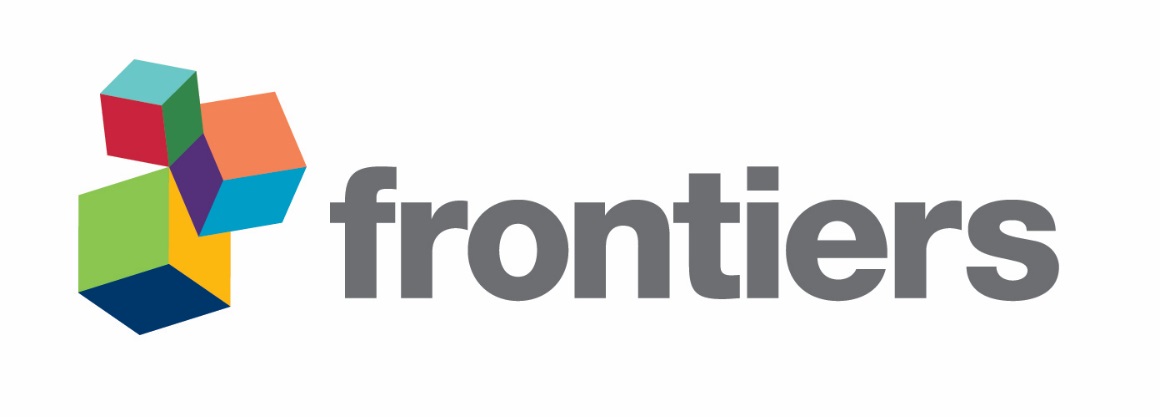
**
