# Supplementary figures and images for: Neuroprotective Effect of Maternal Resveratrol Supplementation in a Rat Model of Neonatal Hypoxia-Ischemia
Source: Front Neurosci. 2021 Jan 15;14:616824. doi: 10.3389/fnins.2020.616824 (PMC7844160; doi:10.3389/fnins.2020.616824)

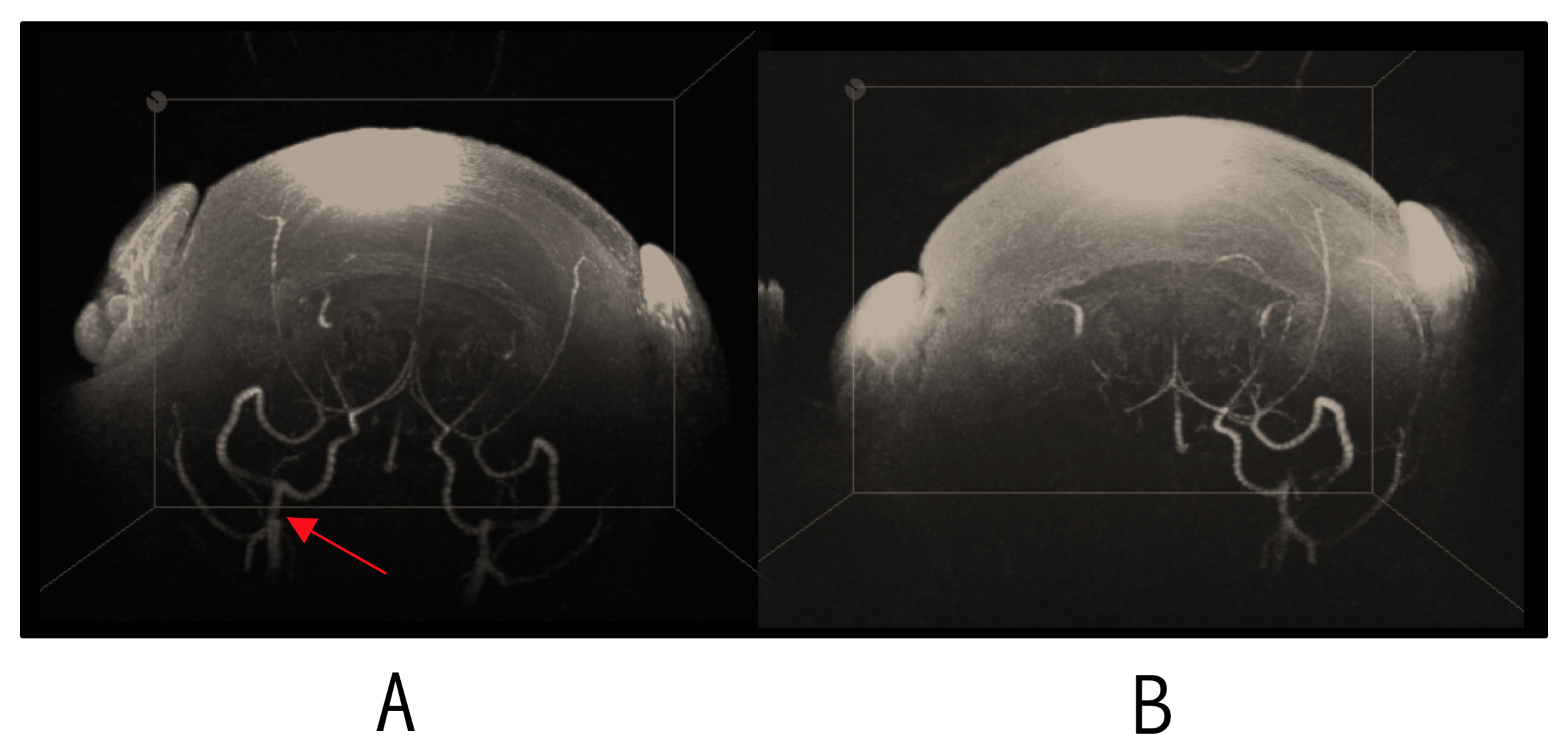

Supplement: Supplementary file 3 [file Image_2.TIFF]

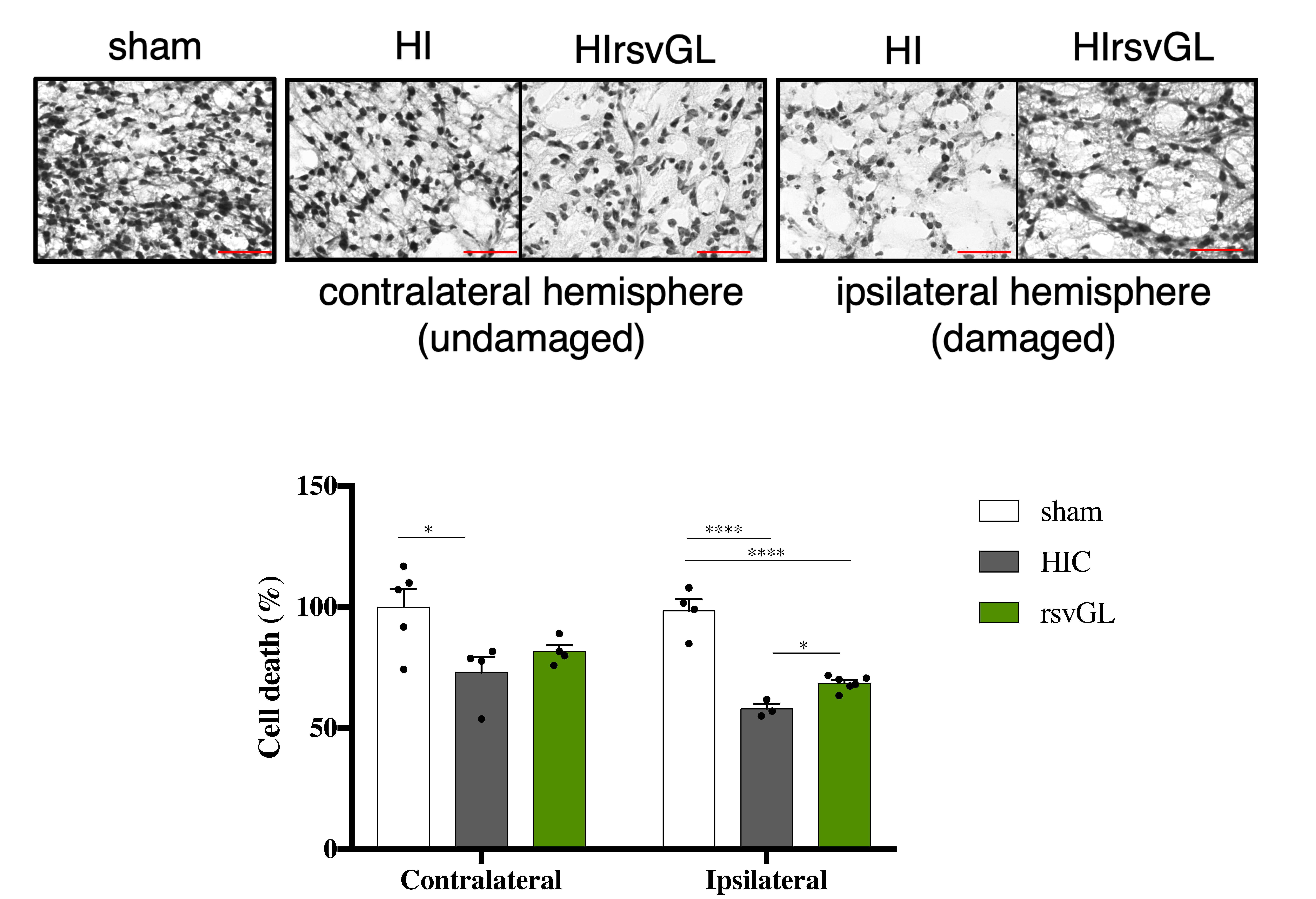

Supplement: Supplementary file 6 [file Image_5.TIFF]
